# Developing an active-learning app to improve critical thinking: item selection and gamification effects

**DOI:** 10.1016/j.heliyon.2021.e08256

**Published:** 2021-10-29

**Authors:** Kota Jodoi, Nobu Takenaka, Satoru Uchida, Shiina Nakagawa, Narahiko Inoue

**Affiliations:** a Graduate School of Integrated Sciences for Global Society, Kyushu University, Fukuoka, Japan; b Faculty of Languages and Cultures, Kyushu University, Fukuoka, Japan

**Keywords:** Critical thinking, Mobile learning, Gamification, Cooperative/collaborative learning, Improving classroom teaching, 21st century skills

## Abstract

Critical thinking (CT) is widely recognized as an important skill and attitude in this modern world, but few apps (web-based or installed on devices) have been developed to effectively train it. There is also little research on what kind of content to put into such apps and in what order, if the content is a series of reasoning questions that are intended as CT exercises. Therefore, this research project, consisting of two studies, tries to demonstrate how exercise questions can be presented to learners to sustain their motivation to work on multiple-choice CT questions. In Study 1, question banks were drawn from popular workbooks for CT and verbal reasoning. The questions were ranked in terms of difficulty based on the participation of university students (N = 73).

In Study 2, the questions were loaded onto two types of web-based apps: (1) one that sequentially gives multiple-choice questions with immediate feedback and (2) one with minimum gamification of group/individual competition. The experiment to examine the effect of the gamification was conducted (N = 114). Both groups with and without gamification showed improvements in the scores of the pre-/post-tests using comparable questions, but there was no clear effect of gamification. These findings show that an effective CT app can be developed using existing question banks but that the effect of gamification needs further research.

## 1 Introduction

### 1.1 Background of research

Critical thinking, CT hereafter, was defined by Robert Ennis (1987), a leading scholar on this topic, as ‘reasonable reflective thinking focused on deciding what to believe or do,’ yet others defined it somewhat differently (Ernest and Patrick, 1991; Paul, 1995). Although there is no single definition of CT, the common consensus is that it is crucial skills in a variety of fields. Butler et al. (2012) reported CT makes positive impacts on real-world outcomes. Also, it has been reported as an important skill in professional contexts, e.g., for nurses, it enables decisions within a short time period (Von Colln-Appling and Giuliano, 2017). The case for using apps for education in CT and argumentation has also been often reported (Easterday et al., 2017; Ismail et al., 2018). Although some factors and processes to design CT apps have been analyzed (Chen et al., 2019), there has been no reporting about how to integrate gamification into an app for CT at the time of this writing.

Gamification is a technique to increase engagement in activities in various fields, such as understanding science efficiently (Sørensen et al., 2016), creating habits of exercise (Hamari and Koivisto, 2015), and managing knowledge (Friedrich et al., 2020). It has also been reported that gamification was useful in educating students on solving problems (Debabi and Bensebaa, 2016; Legaki et al., 2020; Rojas-López et al., 2019).

Against this background, we created a web-based app for students to train themselves on CT in and out of the classroom with gamification. The aim of integrating gamification into the app was to increase the time that students spent on the app so that they would have more opportunities to train their CT skills.

Measuring the effects of training CT is another area about which researchers and educators are concerned. Several CT measurements have been applied, including the Watson-Glaser CT test (Watson and Glaser, 1964) and the Cornell CT test (Ennis et al., 2005), using multiple-choice questions and/or descriptive answer questions. However, these full-fledged tests are not easy enough to use in regular classrooms where time is limited because they take up an entire class meeting (usually 90 minutes in Japanese universities) or beyond. Also, tests written in English are not appropriate for some Japanese students due to their lack of English proficiency, but these tests have not been made available in Japanese as standardized tests. So, we have decided to develop a set of pre-/post-tests to evaluate the effect of our apps on users’ performance in particular areas of CT.

### 1.2 Research questions

We have two major research questions in this study:

(1) How can we prepare a set of questions to present in simple, web-based apps to train students' CT?
(2) Does the use of an app improve students' learning in a particular area of CT?

The first question involves the selection of the content of the apps and installation of certain gamification features in the app. The second question involves the effects of using an app with/without gamification features measured by the experimental and control groups’ pre-/post-test scores. We also examined the ways students used the apps by analyzing the log data of the apps.

This paper consists of two studies that inquire into the research questions above. Study 1 shows how to develop an app to train CT and integrate features of gamification into the app. Study 2 shows the results of the experimental training on students to measure the growth of CT using the app we developed. Following the reports of the two studies, we will then discuss and evaluate the research project and make several suggestions for the future development of apps.

## 2 Study 1: development of the app with gamification

### 2.1 Method

Study 1 took place in 2018 and the first half of 2019 and involved a series of steps to select the contents and determine the design of the app.

#### 2.1.1 Selection of content on the app

The strategy to improve app users' CT is to extract questions from a workbook aiming to improve CT and load them into the app. Users’ CT would be improved by solving these questions from a CT workbook. The characteristic of this app is that gamification is included to help students use it for a longer time and solve more questions, which improves their CT more efficiently.

We reviewed existing textbooks, workbooks, tests of CT and its related areas (argumentation/debate and reasoning/logic), as well as our own expert knowledge as practicing debate educators to make a list of CAN-DO statements (Caena and Punie, 2019, Cambridge University Press, 2019, Heyworth, 2006) to see what specific things students are expected to do in order to be effective critical thinkers. As solving questions in a famous textbook is one way to improve CT (Wallace and Jefferson, 2015), this method was applied in our research. The questions in the book *501 Challenging Logic & Reasoning Problems* (LearningExpress, 2005), a popular workbook to improve CT used in many programs, were chosen as a resource of the contents on the app (Kesselman-Turkel and Peterson, 2004). Although the textbook covers many areas of CT, we extracted six categories shown in Table 1.

**Table 1:** Categories and descriptions shown on the app and the number of questions.

	Description shown on the app	Number of questions
Matching Definitions	In this section, you can train a skill to judge whether a particular situation meets the given conditions. This is, for example, necessary for you to judge whether a particular situation violates the law in your career.	28
Making Judgement	In this section, you can train a skill to correctly and precisely understand situations, conditions, or information when you are given them. This is useful especially when you need to read a lot of documents in your job or research.	24
Verbal Reasoning	In this section, you can train a skill to draw a logically correct conclusion from the given information. This is required to understand the logical flows of discussion.	11
Logic Problems	In this section, you can train a skill to choose a logically correct answer based on a given short passage. These types of questions are used in the written exams of hiring companies.	67
Logic Games	In this section, you can train a skill to choose a logically correct answer based on a given long passage or multiple conditions. These types of questions are also used in the written exams of hiring companies.	35
Analyzing Arguments	This section consists of multiple parts. One of them is a section to train a skill to understand the argument of a given paragraph. Another section asks you to choose the best statement to strengthen or weaken a given argument. These skills are important for you to understand the whole picture of a discussion and draw logically valid claims.	48

#### 2.1.2 Development of gamification features

Since gamification is a buzzword, there is no universal consensus on what gamification means. A general agreement on its sense is that gamification describes the permeation of non-game context such as education with game elements (Schrape, 2014). One of the definitions of gamification is ‘attempts to use the trappings of games (reward structures, points, etc.) to make people engage more with product offerings.’ (Koster, 2005).

Gamification aims to encourage users continue to engage in specific activities for a long time or change their behavior. Typical ways used for gamification include correcting badges (Hamari, 2017; White and Shellenbarger, 2018), sharing a progress chart among users (Domínguez et al., 2013), providing virtual currency (Hamari and Eranti, 2011), and so on.

Gamification has been used in various fields. For example, Hamari (2017) introduced badge-based gamification in a commercial context, and reported a significant trend toward more active use of the service in general, including posting trade proposals, executing trades, and commenting on proposals. In physical activities, people are more likely to exercise by using gamification. Chen and Pu (2014) reported user's physical activities using an app with gamification increased by up to 15 % compared with the controlled group. Recently, many wearable devices have been equipped with gamification. For instance, Apple Watch motivates users by using gamification ‘closing the rings,’ which visualizes the amount of exercise necessary for the day (Davaris et al., 2021).

#### 2.1.3 Gamification in educational context

In the context of education, gamification is used for students to engage in educational activities for a longer time to acquire more educational effects (Brophy, 2013). There are many reports that gamification has positive effects on education (Buckley and Doyle, 2016; Da Rocha Seixas et al., 2016; White and Shellenbarger, 2018). For example, White and Shellenbarger (2018) examined the possibility of using a digital badge to increase user's motivation. Incorporating the system that users can gain badges when they accomplish tasks allows them to engage tasks, bringing more outcomes. Competition-through-ranking system is also often used as gamification to enhance educational effects (Domínguez et al., 2013; Christy and Fox, 2014; Suh and Wagner, 2017). Domínguez et al. (2013) adopted competition as gamification features by showing a progress of each user. They reported that users with gamification got better scores than users who used e-learning materials with no gamification features.

Based on these previously reported findings, our team members brainstormed various features and conferred with an engineer with experience in developing educational apps through several online and face-to-face meetings. After the discussion, four gamification features were selected based on the concept summarized by Toda et al. (2019).

#### 2.1.4 Determining the difficulty of the questions

To realize one of the gamification features, i.e., ordering the questions from easier to more difficult (see Section 2.2.2), the questions on the app were organized in terms of the specific skills tested and the difficulty of the questions. First, 213 questions were chosen by our research team from *501 Challenging Logic & Reasoning Problems* (LearningExpress, 2005), and these questions were divided into the six categories shown in Table 1 based on the original book's descriptions with minor modifications.

Next, the difficulties of the questions were determined by testing them on the students. For the experiment to measure the correct answer rates of each question, 213 questions were divided into seven pools (P1–P7) and first-year undergraduate students from University A (a relatively small college in the western part of Japan) and undergraduate students in the English Speaking Society (ESS) of University B (a relatively large university in the same region) answered them in April 2019. 54 students from University A answered one pool each and 19 students from University B answered two pools each (Table 2).

**Table 2:** Number of questions and respondents from university A and B to each question set.

	P1	P2	P3	P4	P5	P6	P7	Total
Number of questions	31	30	32	32	28	30	30	213
Respondents from university A	10	8	8	7	7	7	7	54
Respondents from university B	6	5	5	7	5	5	5	38

### 2.2 Results

#### 2.2.1 Selection of the content

We considered various content for our CT apps in terms of typicality, popularity, exercise question types (multiple-choice or open-ended), availability of sample answers with explanations, etc. We also considered the prospect of deriving our original content from arguments used in competitive debating. We chose three types of content that will be eventually integrated: (1) simpler logic and reasoning problems mostly from existing question banks (LearningExpress, 2005); (2) a variety of problems based on a popular workbook on arguments (Morrow and Weston, 2019); and (3) problems based on arguments often found in competitive debating, which will be created by student debaters and debate educators. The questions in (1) and (2) were translated from English into Japanese and checked by multiple researchers on our team. The aim of selecting these three different types of questions was that users could learn CT step by step. Type (1), called ‘501,’ includes relatively easy question sets to solve, so we determined it would be the first step before the challenging Type (2) workbook and Type (3) debate-related questions.

#### 2.2.2 Gamification features

We reviewed the gamification features that had been reported at the start of our research project and discussed what should be included in the app as our basic strategy was to increase the efficacy of CT training, by increasing engagement. The gamification features for increasing engagement were listed, and the appropriate contents for the app were chosen considering the user interface and possible functions of the app and the teaching experiences of our research teams. Among the elaborated gamification elements for education (see for instance, Toda et al., 2019), we decided to integrate four features of gamification into this app that were reported to increase engagement. These were:

1. Placing questions from easy to difficult so that users did not give up at the very first stage.
2. Creating groups of three or four students and showing the rankings of the group's progress, which were calculated as the average of the students' scores within a group (Figure 1).

**Figure 1:**
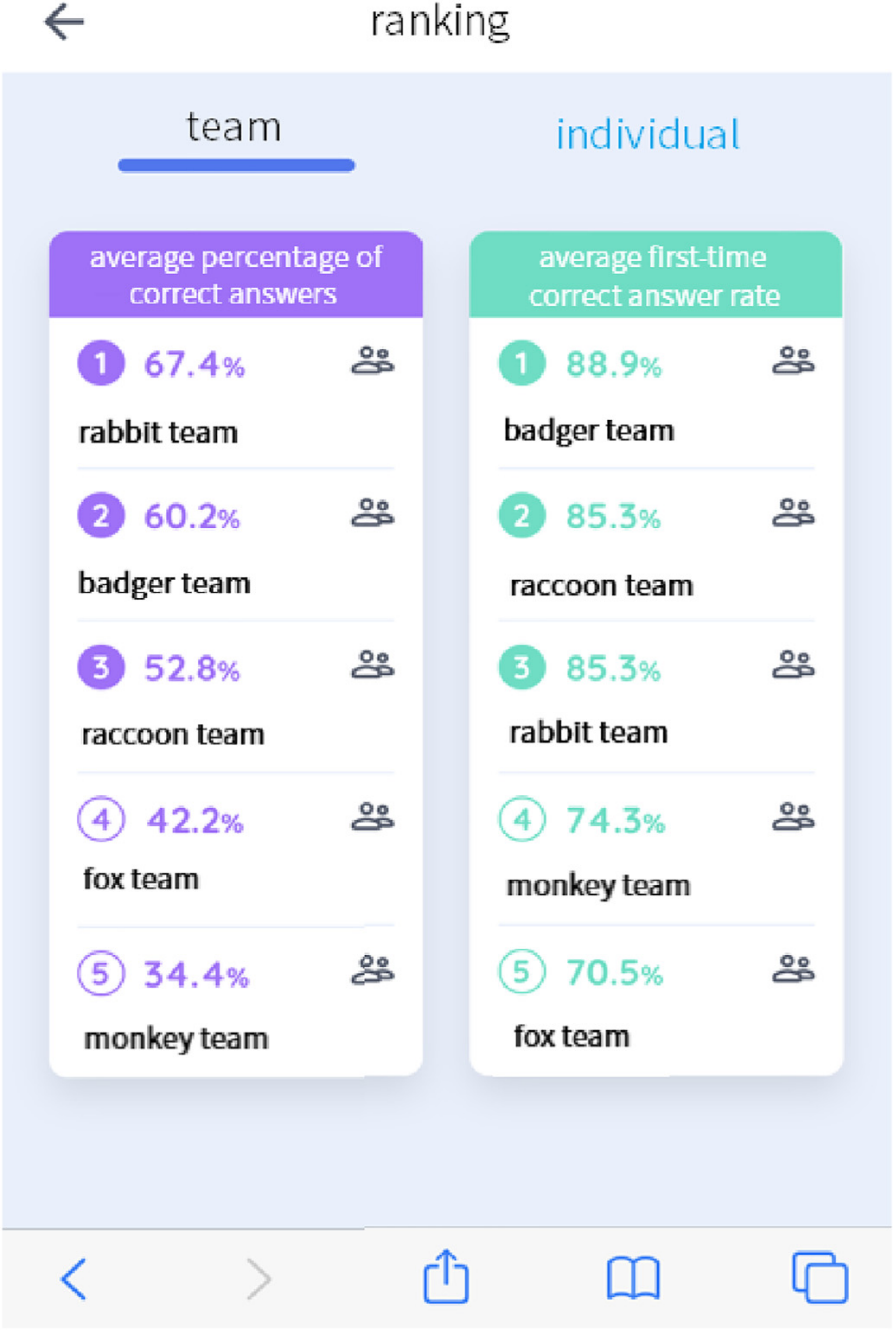
Progress of each team (left) and correct answer rate in the first trial of each group (right). All of them are calculated as the average scores of students in each group.
3. Showing the ranking of each student's individual progress (Figure 2).

**Figure 2:**
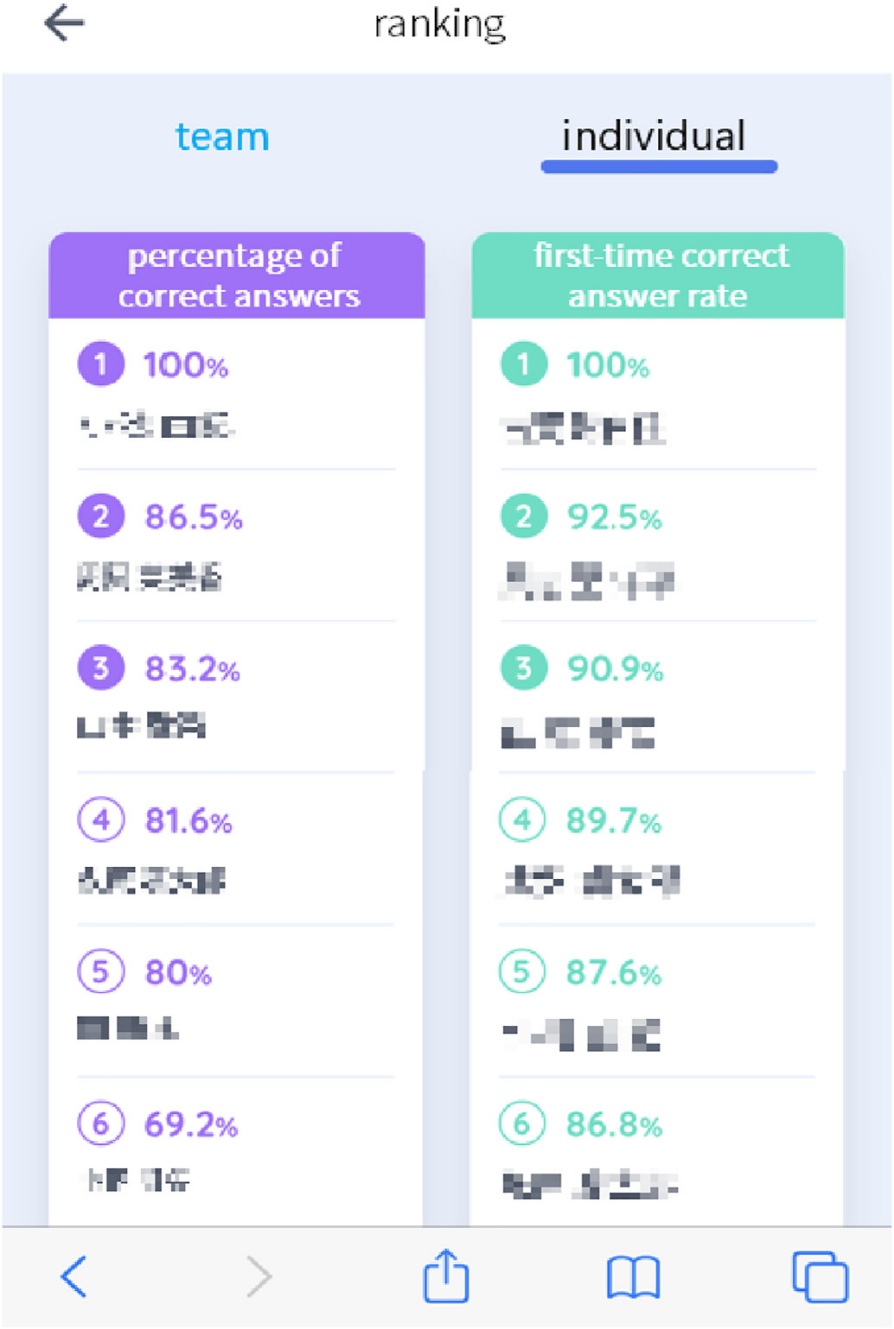
Progress of individual students (left) and correct answer rate in the first trial of students (right). Students' names were masked to protect personal information.
4. Limiting the maximum sets of questions to answer per day. Users could answer up to three sets, each of which consisted of three or four questions.

The concepts we adopted for these features were 1: Level, 2: Competition, Cooperation, 3: Stats, and 4: Rarity (Toda et al., 2019). For the first gamification features, Toda et al. (2019) reported hierarchical layers are important for users to increase engagement. To make hierarchy in the app, easy questions, which were assumed most students are easy to make correct marks, were put in the first stage, and the level of questions get gradually difficult. For the second gamification feature, since the concepts of Competition and Cooperation were two or more players collaborating and achieving a common goal would increase engagement, we created some groups consisting of three or four students. For the third gamification feature, we show the users’ progress because of the Stats concept, where visible information within the app would increase engagement. The purpose of the last gamification feature was Rarity, where limited resources in the app contribute to engagement. The number of the maximum sets were decided based on the interview with students. The effect of each gamification features was discussed in the latter section.

#### 2.2.3 Determining the difficulty of the questions

**Figure 3:**
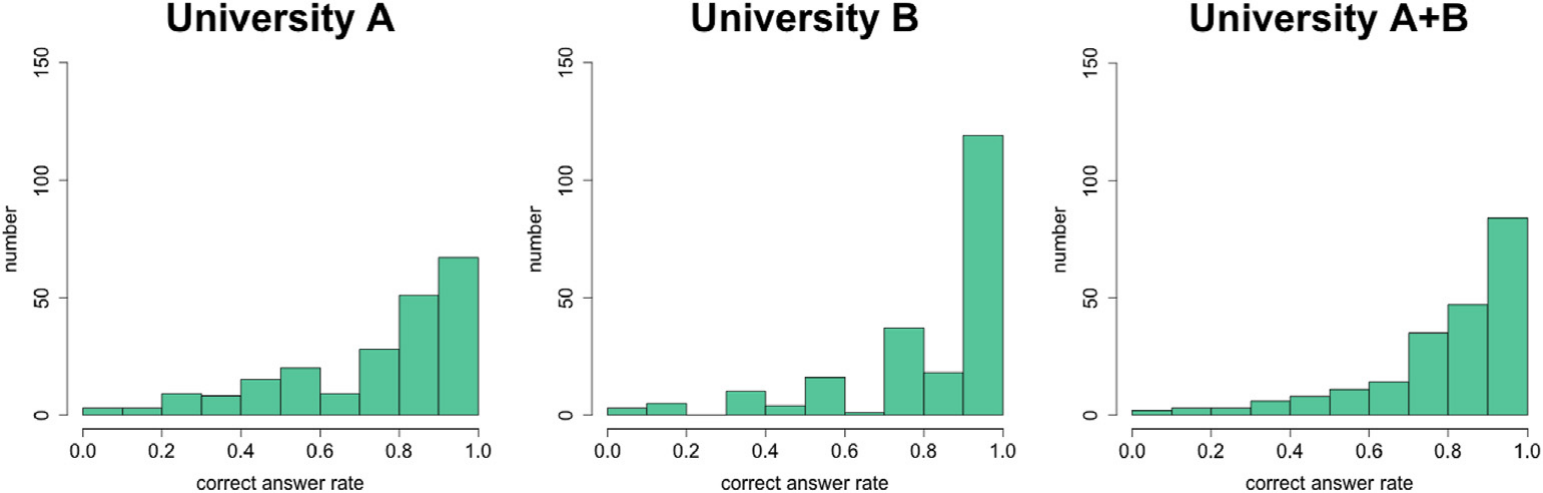
Histograms for correct answer rates for University A, University B, and combined group in order to rank questions from *501 Challenging Logic & Reasoning Problems*.

A total of 213 questions were answered by students from University A and University B; then the correct answer rates for each question were calculated. Average, maximum, and minimum correct answer rates and standard deviations are shown in Table 3, and the histograms are shown in Figure 3. A t-test yielded significant differences between the two groups of students (p < 0.05, t = 4.14). We decided to use the average correct answer rates of all the students to rank the questions in terms of general difficulty for the undergraduate students of the two universities, from which the participants for Study 2 were going to be derived.

**Table 3:** Average, maximum, and minimum answer rates and standard deviations (S.D.) of university A and university B for the ranking test of questions drawn from *501 Challenging Logic & Reasoning Problems*.

	Correct answer rate			S.D.
	Average	Maximum	Minimum	
University A	0.76	0.91	0.45	0.10
University B	0.85	1.00	0.61	0.08
A & B combined	0.79	1.00	0.45	0.10

Descriptive statistics for the questions in each category are shown in Table 4. The questions were loaded onto the apps based on the difficulty ranking (average correct answer rates) in each category, to be presented to the users from the easiest to the most difficult in a sequence in each category. In the app with gamification, the questions were grouped in sets of 3–5 questions each; the easiest set was presented to the users first with increasing difficulty.

**Table 4:** Average answer rates for each category of university A and university B combined in the ranking test of questions from *501 Challenging Logic & Reasoning Problems*.

	# of Questions	Average Correct	Max.	Min.	S.D.
Matching Definitions	28	0.79	1.00	0.10	0.20
Making Judgement	24	0.81	1.00	0.16	0.20
Verbal Reasoning	11	0.70	1.00	0.21	0.26
Logic Problems	67	0.82	1.00	0.00	0.20
Logic Games	35	0.88	1.00	0.31	0.17
Analyzing Arguments	48	0.74	1.00	0.20	0.19
Total	213	0.79	-	-	0.20

### 2.3 Discussion

We found that there were enough questions in each question type with varied difficulty for the undergraduate students from the two universities from which we planned to recruit the participants of Study 2. Although there were some differences between the two groups of students in this study, the average correct answer rates gave us approximate difficulty levels of the questions. Presenting questions in the app from easier to more difficult would ensure the users started from easier questions to gain confidence to move on to more difficult ones.

## 3 Study 2: experiment to measure the growth of CT

### 3.1 Method

#### 3.1.1 Participants

We used three slightly different groups, A, B, and C in this experiment. Our basic strategy is to make experimental and control groups within the available student groups we had access to, in order to examine the gamification effects and app use. More specifically, Group A consisted of freshmen at University A, who were divided into two subgroups (A-1 and A-2). Those in Group A-1 used an app with gamification, and those in Group A-2 used an app without gamification; the questions in both apps were the same. Group B consisted of freshmen at University B, and they were divided into three subgroups (B-1, B-2, and B-3). Those in Group B-1 used the app with gamification, those in Group B-2 used the one without gamification, and Group B-3 did not use any app or work on our CT questions at all. Group C consisted of people who had experience with debate activities, and the group was divided into two subgroups (C-1 and C-2). Those in C-1 used the app with gamification, and those in C-2 did not use any app (Table 5).

**Table 5:** Number of students and conditions of each group for the experiment.

	Group A		Group B			Group C	
	A-1	A-2	B-1	B-2	B-3	C-1	C-2
Number of students	24	27	15	11	26	12	9
App use	Yes	Yes	Yes	Yes	No	Yes	No
Gamification	Yes	No	Yes	No	N.A.	Yes	N.A.

Those in Group A and Group B were from the undergraduate freshmen class of a Japanese university, so most of them were 18 or 19 years old. Those in Group C included people of different ages from 18 to 25. Given the nature of the existing groups, the grouping is not completely randomized but the respective subgroups in Groups A, B, and C do not show any significant difference in the pre-test results.

#### 3.1.2 Materials (differences between the app with and without gamification)

The two types of CT apps developed in Study 1 were used in all experimental groups. The total number of questions available on the apps was 187, i.e., 26 less than the original pool since those questions were used in the pre-/post-tests (Table 6). The questions were presented to users in random order within each category in the app without gamification and in the order of difficulty in the app with gamification. For the app with gamification, gamification features described in Section 2.2.2 were incorporated, while the app without gamification has no gamification feature.

**Table 6:** Number of questions in each category for the apps.

	Original number of questions	Questions used for pre-/post-tests	Questions on the apps
Matching Definitions	28	2	26
Making Judgement	24	4	20
Verbal Reasoning	11	2	9
Logic Problems	67	6	61
Logic Games	35	6	29
Analyzing Arguments	48	6	42
Total	213	26	187

#### 3.1.3 Procedures

Students were required to use the respective app for two months, except for the students in control groups (B-3 and C-2). In Groups A-1, A-2, B-1, and B-2, the instructor distributed brief written instructions in class, including an incentive to use the app, i.e., a maximum of 10% bonus points toward the final grade. The students were enrolled in required EFL academic courses with the primary content being debating in English. The use of the app was justified as part of learning CT, especially reasoning skills, which are closely related to debating. There were no related classroom activities or mentions of the app by the instructor unless students asked questions, which turned out to be rare. The ESS group participants (C-1) also used the app at their own pace outside their regular debate activities. Only once was an email notification sent to all the users to let them know the progress of the app at the mid-point of the experimental, i.e., app use, period.

#### 3.1.4 Data collection

##### 3.1.4.1 Pre-test and post-test

Based on the results of the ranking test in Study 1, we designed the pre-/post-tests to measure CT skills. Questions for each test were chosen considering the correct answer rates so that the total values of the pre-/post-tests were almost the same. This process was manually manipulated with several trials to choose the questions to include in two versions of the test. We also paid attention to additional features of the questions, e.g., the length of the question passage, so that the two tests looked similar on paper.

The results from determining the difficulty of each question allowed us to select questions for the pre-/post-tests out of the whole pool of questions. The first step was to choose questions from each category shown in Table 1. We chose at least one question from each category. The number of questions for the pre-/post-tests are shown in Table 7. The second step was to calculate the correct answer rates of the chosen questions in order to make sure that the total value of each test had almost the same difficulty. The correct answer rates of the questions for the pre-/post-tests are shown in Table 8. The average correct answer rate of the pre-test was 0.707 and that of the post-test was 0.701, which showed we successfully designed the pre-/post-tests.

**Table 7:** Number of questions in each category for pre-/post-tests.

	Pre-test	Post-test
Matching Definitions	1	1
Making Judgement	2	2
Verbal Reasoning	1	1
Logic Problems	3	3
Logic Games	3	3
Analyzing Arguments	3	3
Total	13	13

**Table 8:** Correct answer rates of each question in pre-/post-tests (13 questions each) Drawn from Ranking Experiment.

	1	2	3	4	5	6	7	8	9	10	11	12	13	Total
	A	B	C	D	D	D	E	E	E	F	F	F	F	
pre	0.62	0.50	0.62	0.60	0.80	0.42	0.85	0.85	0.85	0.71	0.62	0.87	0.75	0.707
post	0.57	0.87	0.71	0.71	0.42	0.57	0.90	1.00	0.90	0.37	0.57	0.71	0.85	0.701

*Notes*.

1. A: Matching Definitions, B: Making Judgement, C: Verbal Reasoning, D: Logic Problems, E: Logic Games, F: Analyzing Arguments.

2. The total score is calculated as an average score of question from 1 to 13.

The validity of the pre-/post-tests was ensured by choosing the questions from each of the six categories of the original test bank of 213 questions as shown in Table 7. The tests’ content validity was confirmed by measuring the change in the users' answers to the types of questions they practiced using the apps. The original question bank had a certain level of validity because it represented typical CT skill questions often practiced in existing textbooks, workbooks, and tests that we reviewed as we explained in 2.2.1.

To check the test-retest reliability of the pre-test, a university class consisting of students from the same department was randomly assigned to two groups (N = 27, 26), and the scores of the pre-test were measured. The result of the t-test showed there was no statistical difference between the two groups (t = 2.00, p = 0.82). This allowed us to conclude that the tests had the reliability. As the post-test was designed in the same way, the same result could be applied to the post-test.

Pre-/post-tests were carried out for Groups A, B, and C. Before starting to use the app, the pre-test was conducted for all groups' students (A-1, A-2, B-1, B-2, B-3, C-1, and C-2) around October 2019. After the experimental period, the post-test was carried out for all groups around January 2020. Although these tests don't measure their whole CT skills, it is expected that they can measure the growth of the CT skills related to this study by comparing the score of pre-/post-tests.

##### 3.1.4.2 Survey on app use

Together with the post-test, we surveyed the app use for all groups. We asked the ‘gamification’ group to evaluate the gamification of the app. Table 9 shows the questions on gamification. All questions are available in Appendix 2.

**Table 9:** Questions on the survey after using the app.

	Question
Q1	Did you think the limit on the question sets you could answer being up to three was large or small?
Q2	Did the comparison with other groups in the first trial of average percentage of correct answers and average first-time correct answer rates increase your motivation to use the app?
Q3	Did the comparison with other users in the first trial of average percentage of correct answers and average first-time correct answer rates increase your motivation to use the app?

##### 3.1.4.3 Logs of app use

The records on the users’ use of the app were preserved on the central management page of the website, to which only teachers had access. They include individual progress and correct/incorrect response records for each question, among other information. Those data are downloadable as a CSV file to analyze from a number of viewpoints.

### 3.2 Results

#### 3.2.1 Pre-test and post-test

We observed an increase of average correct answer rates at the pre-/post-tests in groups that used the app with gamification and without it (Table 10). All experimental groups increased the mean by more than 8%, while the average correct answer rates of the control groups (B-3 and C-2) stayed about the same between the two tests. A paired t-test was also conducted with all groups, which showed significant differences between the pre-/post-tests for four out of the five experimental groups, while the control groups showed no significant difference.

**Table 10:** Mean and standard deviation (S.D.) of pre-/post-tests of groups that used the app.

	Gamification	Mean of correct answer rate			S.D.		t-test	
		Pre	Post	Change	Pre	Post	t-value	p-value
A-1	Yes	0.795	0.888	0.093	0.143	0.086	2.819	<0.05
A-2	No	0.769	0.876	0.107	0.160	0.107	3.050	<0.05
B-1	Yes	0.769	0.907	0.138	0.146	0.089	3.641	<0.05
B-2	No	0.821	0.960	0.139	0.057	0.040	5.982	<0.05
B-3	N.A.	0.784	0.837	0.053	0.141	0.115	1.662	0.108
C-1	Yes	0.821	0.904	0.083	0.122	0.089	1.766	0.105
C-2	N.A.	0.855	0.889	0.034	0.084	0.089	1.272	0.239

#### 3.2.2 Questionnaire survey on app use

A questionnaire survey was conducted with all groups after the post-test (N = 154). Questions on the gamification of the app were asked only to the experimental groups (A-1, B-1, and C-1). Results to the questions in Table 9 are shown in Tables 11, 12, and 13.

**Table 11:** Answers to the Question “Did You Think the Limit on the Question Sets You Could Answer Being Up to Three was Large or Small? Answer in the Range of 1 (Small) to 5 (Large)”.

	1 (small)	2	3	4	5 (large)
A-1	4	11	10	0	0
B-1	6	8	1	0	0
C-1	2	8	2	0	0

**Table 12:** Answers to the question: “Did the comparison with other groups in the first trial of average percentage of correct answers and average first-time correct answer rates increase your motivation to use the app?”

	Average percentage of correct answers					Average first-time correct answer rate				
	1	2	3	4	5	1	2	3	4	5
A-1	3	3	9	5	5	3	3	9	6	4
B-1	3	4	3	2	3	2	5	4	3	1
C-1	0	2	3	3	4	1	3	4	0	4

*Note*. The choices were in the range of 1 (not incentivized at all) to 5 (strongly incentivized). N (A-1) = 25, N (B-1) = 15, N(C-1) = 12.

**Table 13:** Answers to the question: “Did the comparison with other users in the first trial of average percentage of correct answers and average first-time correct answer rates increase your motivation to use the app?”

	Average percentage of correct answers					Average first-time correct answer rate				
	1	2	3	4	5	1	2	3	4	5
A-1	1	1	5	7	11	1	1	6	7	10
B-1	1	5	4	2	3	0	3	9	2	1
C-1	1	4	3	2	2	1	5	3	1	2

*Note*. The choices were in the range of 1 (not incentivized at all) to 5 (strongly incentivized). N (A-1) = 25, N (B-1) = 15, N(C-1) = 12.

#### 3.2.3 Logs of app use

Downloading the logs of Groups, A-1, A-2, B-1, and B-2 and analyzing them enabled us to calculate the number of total answers and the progress of each group (Table 14). The average number of answers per user was larger than the number of total questions on the app because if users chose a wrong answer, they would have to try the question again to proceed to the next question. Also, since the ‘Progress (%)’ was calculated based on correct answer rates, it does not correlate with the total number of answers per user, which includes wrong answers.

**Table 14:** Total number and average of questions users answered and their progress.

	Gamification	Number of students	Total number of questions	Total answers	Answers/one user	Progress (%)
A-1	Yes	24	187	6652	277	70.1
A-2	No	27	187	7374	273	87.1
B-1	Yes	15	187	2875	191	96.3
B-2	No	12	187	2803	233	86.4

### 3.3 Discussion

#### 3.3.1 Training effect on CT by using the app

Our expectation about the effect of training on CT was that by using the app with gamification, users would focus on it for a longer time; thus, their scores would increase more compared to users who used the app without gamification. Significant changes in CT were observed for A-1, A-2, B-1, and B-2 after using the app (Table 10). Although we could not find a significant change statistically for Group C-1, the mean of correct answer rates increased by 0.083, which was almost the same as with other groups. These results reveal that there is a positive correlation between using the app and improving CT.

One of the main goals of our experiments was to find some effects of gamification in the app. When we compared the correct answer rates before and after using the app for A-1 (with gamification) and A-2 (without gamification), there was no significant difference between them. This tendency is the same as that between B-1 (with gamification) and B-2 (without gamification). These results did not support our prediction. To investigate the reasons for these results, further analysis of users’ behaviors was conducted, which will be discussed in the next section.

#### 3.3.2 Effect of gamification on the app

Table 14 shows there was no significant difference in the number of answers per user between groups with and without gamification. This led us to conclude that gamification on the app did not achieve our purpose to promote continuous use of the app. Namely, since users used the app even without gamification and practiced the questions, there was no significant difference between their scores in the two conditions—with/without gamification. The survey of users who used the app with gamification revealed why gamification on the app did not work as expected. To the question of whether the limitation of answerable questions up to three was unnecessarily restricting students' app use, the majority answered that the limit was too restrictive (Table 12). The results demonstrate that our limitation on the app was too strict for users, and rather than functioning as an incentive to regularly use the app, users might have lost some opportunities to use the app more. This indicates that the limitation of answerable sets should be relaxed to more than three. On the other hand, some users of the app without gamification obviously crammed to work on the app on a few days before the post-test, which may have artificially increased their scores on the post-test. We should find optimal limitations to foster users’ regular use while accommodating their differences in available time for app use.

In order to promote the users' progress through competition and cooperation, we installed the function to rank the progress of users and groups (Figure 1 and Figure 2). A survey was conducted to ask users whether these rankings worked to sustain motivation to use the app (Table 12 and Table 13). These results indicate competition among individuals rather than groups increased their sustained motivation. In particular, 44% of users of Group A-1 answered they were ‘strongly incentivized’ by the competition of average percentage of correct answers among individuals (Table 13). On the other hand, there was no remarkable difference between the effect of group and user competitions for Group B-1. That might be because there were not enough users to engage in competition in Group B-1.

## 4 Conclusion

We have created a novel app to train CT using gamification that focuses on sustaining users' motivation by ordering questions according to their difficulty, introducing a ranking system of average percentage of correct answers, and setting a limitation on answerable questions in a day (Study 1). In Study 2, we conducted an experiment to examine the gamification effect on the app developed in Study 1. Although we found a few things that should be fixed, a significant improvement in users’ CT through using the app in both groups with gamification and without gamification was observed.

As far as we know, there has been no empirical research to compare the effects of gamification on the use of web-based apps to improve the CT skills of university students. The results suggest that the app would be useful not only for students but also for instructors because it can provide additional exercises to reinforce classroom study/activities and allow the instructor to use the limited classroom time on activities that require face-to-face interaction.

Through the use of this app, students would have opportunities for active learning, although such generalization requires a more robust experimental design to control various intervening factors. Active learning is a broad word, and one of its core meanings is that students continue to learn positively not passively. This app contributed to providing our students the experience to train themselves in CT by with gamification. The results showed many students did engage in this app and were satisfied with their learning.

Further updates for the app will be conducted based on the results of the experiments and users’ feedback. First, we plan to increase the number of answerable questions in a day since the majority of the users answered that the limitation in this experiment was too restrictive. Second, we will send more frequent notifications. In this experiment, a notification was sent only once, but many users thought it was not enough to remind them to engage in the app continuously. A system to send notifications to users who do not make good progress should also be considered. These attempts will optimize the app and achieve more efficient CT education.

## Declarations

### Author contribution statement

Kota Jodoi: Conceived and designed the experiments; Performed the experiments; Analyzed and interpreted the data; Contributed reagents, materials, analysis tools or data; Wrote the paper.

Nobu Takenaka: Conceived and designed the experiments; Analyzed and interpreted the data; Contributed reagents, materials, analysis tools or data.

Satoru Uchida: Conceived and designed the experiments.

Shiina Nakagawa: Conceived and designed the experiments; Contributed reagents, materials, analysis tools or data.

Narahiko Inoue: Conceived and designed the experiments; Analyzed and interpreted the data; Contributed reagents, materials, analysis tools or data; Wrote the paper.

### Funding statement

This work was supported by JSPS KAKENHI Grant Number JP18H01055; Kyushu University QR Program Tsubasa Project, Japan.

### Data availability statement

Data will be made available on request.

### Declaration of interests statement

The authors declare no conflict of interest.

### Additional information

No additional information is available for this paper.
