# Supplementary material for: Developing an active-learning app to improve critical thinking: item selection and gamification effects
Source: Heliyon. 2021 Oct 29;7(11):e08256. doi: 10.1016/j.heliyon.2021.e08256 (PMC8571479; doi:10.1016/j.heliyon.2021.e08256)
Supplement: supplementary [file mmc1.docx]

**Appendix 1**

Sample CT Questions on the Apps

Sample questions and choices on the app for each section are below.

| Section | Sample question and answer choices |
| --- | --- |
| Matching Definitions | **Violating an apartment lease** occurs when a tenant does something prohibited by the legally binding document that he or she has signed with a landlord. Which situation below is the best example of violating an apartment lease?   1. Tim has decided to move to another city, so he calls his landlord to tell him that he is not interested in renewing his lease when it expires next month. 2. Valerie recently lost her job and, for the last three months, has neglected to pay her landlord the monthly rent they agreed upon in writing when she moved into her apartment eight months ago. 3. Mark writes a letter to his landlord that lists numerous complaints about the apartment he has agreed to rent for two years. 4. Leslie thinks that her landlord is neglecting the building in which she rents an apartment. She calls her attorney to ask for advice. |
| Making Judgement | The owner of the Sun Times Chevrolet dealership tells Officer Chervenak that someone has been stealing running boards and other parts off of the vans he has parked in the south lot sometime after 10:30 p.m. Officer Chervenak decides to patrol the area carefully.  Which of the following situations should she investigate?   1. After midnight, a man in his early twenties is walking up and down rows of new pickups parked near the edge of the dealership. 2. After midnight, a panel truck pulls out of the vacant lot next to the dealership near where the vans are lined up. 3. After midnight, two youths in baggy pants and t-shirts are rollerblading in and out of the new cars on the Sun Times lot. 4. After midnight, a station wagon drives into the lot and stops near the door to the main showroom. A man gets out and starts unloading a mop, a bucket, and a broom. |
| Verbal Reasoning | Look for the statement that must be true according to the given information.  On January 4, 1998, the city of Mitchellville recorded its lowest temperature since 1896. The temperature in Mitchellville on January 4 was 5 degrees Fahrenheit. Two days earlier, two inches of snow fell on the city, and this, too, was a 102-year-old record.   1. The temperature in Mitchellville has never reached 4 degrees Fahrenheit. 2. Deep snow in Mitchellville is extremely rare. 3. Temperatures were not recorded in Mitchellville prior to 1896. 4. Mitchellville has mild winters and mild summers. |
| Logic Problems | ・All the trees in the park are flowering trees.  ・Some of the trees in the park are dogwoods.  ・All dogwoods are flowering trees.  If the first two statements are true, the third statement is   1. TRUE 2. FALSE 3. Uncertain |

|  |
| --- |
|  |

| Logic Games | Answer questions 1 through 3 on the basis of the information below.  ==========  Five roommates—Randy, Sally, Terry, Uma, and Vernon—each do one housekeeping task—mopping, sweeping, laundry, vacuuming, or dusting—one day a week, Monday through Friday.  •Vernon does not vacuum and does not do his task on Tuesday.  •Sally does the dusting and does not do it on Monday or Friday.  •The mopping is done on Thursday.  •Terry does his task, which is not vacuuming, on Wednesday.  •The laundry is done on Friday and not by Uma.  •Randy does his task on Monday.  ==========  Question 1: When does Sally do the dusting?   1. Friday 2. Monday 3. Tuesday 4. Wednesday 5. Thursday   Question 2: What task does Terry do on Wednesday?   1. Vacuuming 2. Dusting 3. Mopping 4. Sweeping 5. Laundry   Question 3: On what day is the vacuuming done?   1. Friday 2. Monday 3. Tuesday 4. Wednesday 5. Thursday |
| --- | --- |
| Analyzing Arguments | Originating in the 1920s, the pyramid scheme is one of the oldest con games going. Honest people are often pulled in, thinking the scheme is a legitimate investment enterprise. The first customer to ‘fall for’ the pyramid scheme will actually make big money and will therefore persuade friends and relatives to join also. The chain then continues with the con artist who originated the scheme pocketing, rather than investing, the money. Finally, the pyramid collapses, but by that time, the scam artist will usually have moved out of town, leaving no forwarding address.  This paragraph best supports the statement that   1. It is fairly easy to spot a pyramid scheme in the making. 2. The first customer of a pyramid scheme is the most gullible. 3. The people who set up pyramid schemes are able to fool honest people. 4. The pyramid scheme had its heyday in the 1920s, but it’s making a comeback. 5. The pyramid scheme got its name from its structure. |

**Appendix 2**

Survey Form on App Use

(1) and (3) were answered by all students, and (2) was answered only by the students who used the app with gamification.

(1) About the questions on the app, answer the questions below.

1.1 Level of difficulty

easy 1 2 3 4 5 difficult

1.2 Was using the app interesting?

boring 1 2 3 4 5 interesting

1.3 Do you want to challenge the questions on the app in the future?

1. Strongly agree

2. Agree

3. Neither

4. Disagree

5. Strongly disagree

1.4 Do you think it is better to practice these questions?

1. Yes

2. No

3. Not sure

If you answer 1 or 2, why?

1.5 How do you want to solve these questions in the future?

1. on paper or in a book

2. on a smartphone

3. on a laptop

4. other

1.6 Have you ever practiced these questions before this experiment?

1. Yes

2. No

3. Not sure

If you answer 1, where?

1.7 When you challenge these questions in future, how do you feel?

1. Positive

2. A little positive

3. Neither

4. A little negative

5. Negative

(2) Did you think the limit on the question sets you could answer being up to three was large or small?

2.1 What do you think about the limitation of answerable questions up to three?

too small 1 2 3 4 5 too much

2.2 Did you read the explanations for skills on the app? And did they incentivize your study on the app?

Read the explanations?

Not at all 1 2 3 4 5 Read all of them

Incentivized?

Not at all 1 2 3 4 5 Incentivized very much

2.3 Did you see the individual rankings?

Ranking of the progress rate

Not at all 1 2 3 4 5 Saw sometimes

Ranking of the correct answer rate in the first trial

Not at all 1 2 3 4 5 Saw sometimes

2.4 Did the comparison of rankings with other students incentivize your study on the app?

Comparison of the progress rate

Not at all 1 2 3 4 5 Incentivized very much

Comparison of the correct answer rate in the first trial

Not at all 1 2 3 4 5 Incentivized very much

2.5 Did you see the group rankings?

Ranking of the progress rate

Not at all 1 2 3 4 5 See sometimes

Ranking of the correct answer rate in the first trial

Not at all 1 2 3 4 5 See sometimes

2.6 Did the comparison of rankings with other groups incentivize your study on the app?

Comparison of the progress rate

Not at all 1 2 3 4 5 Incentivized very much

Comparison of the correct answer rate in the first trial

Not at all 1 2 3 4 5 Incentivized very much

2.7 Did the making of groups on the app incentivize your study?

Not at all 1 2 3 4 5 Incentivized very much

(3) Any comments on the app
